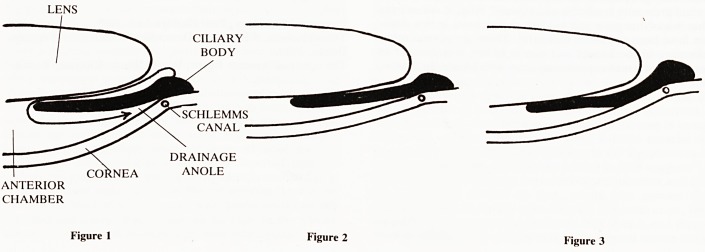# Soap Gets in Your Eyes

**Published:** 1990-12

**Authors:** Alistair Laidlaw, Philip Bloom

**Affiliations:** Bristol Eye Hospital, Lower Maudlin Street, Bristol; Bristol Eye Hospital, Lower Maudlin Street, Bristol

## Abstract

We present a previously unreported series of five cases of acute angle closure glaucoma associated with watching the Australia soap opera "Neighbours". Two cases were bilateral and associated with watching two episodes of "Neighbours" on the same day. The pathogenesis, and possible role of watching soap operas in the causation of primary angle closure glaucoma is discussed.


### Soap Gets in Your Eyes

**Alistair Laidlaw FRCS Glas**
**Philip Bloom FRCS Ed**
Bristol Eye Hospital, Lower Maudlin Street, Bristol

**Abstract**
We present a previously unreported series of five cases of acute angle closure glaucoma associated with watching the Australia soap opera "Neighbours". Two cases were bilateral and associated with watching two episodes of "Neighbours" on the same day. The pathogenesis, and possible role of watching soap operas in the causation of primary angle closure glaucoma is discussed.

**Clinical Series**

Five patients, four female and one male, aged from fifty nine to seventy three years, presented to the casualty department of Bristol Eye Hospital in the two weeks preceding Christmas 1989 with primary angle closure glaucoma.

Three of the patients had unilateral angle closure. These three volunteered that their symptoms had started following that evenings' episode of BBC Televisions' soap opera "Neighbours".

The other two patients described asynchronous but sudden onset of bilateral ocular pain with misting of vision. These patients stated that their symptoms arose on a day when they had watched both episodes of Neighbours, which is screened at lunchtime and again in the early evening.

**Discussion**

Primary angle closure glaucoma typically presents in middle aged to elderly, predominantly female adults. Symptoms include sub-acute onset of aching pain, reddening of the globe, misting of vision, haloes around lights and occasionally nausea and vomiting.

Signs are decreased visual acuity, intense reddening of the sclera, a hazy dull cornea and an oval, mid-dilated and unreactive pupil.

A family history can often be elicited and the patient may have suffered mild attacks in either eye over the preceeding few weeks. Affected patients are usually long sighted. The normal flow of aqueous (Fig 1) occurs from the ciliary body in the posterior chamber through the pupil between the lens and iris, to the anterior chamber and thence to the drainage angle. In a deep anterior chamber there is little iris-lens contact.

Angle closure typically occurs in eyes with shallow anterior chambers, as are found in the short eyes of hypermetropic people, the tendency to which is inherited. Females usually have shallower anterior chambers than males and due to lens growth the anterior chamber becomes shallower with age. A mid-dilated pupil may occur in the dark or with heightened emotion, such as can be experienced whilst watching television soap operas. This induces considerable iris-lens contact (Fig 2), which prevents the normal flow of aqueous from the posterior to the anterior chamber by acting as a 'bottleneck'. In predisposed eyes the resulting iris bombé closes the drainage angle before the iris-lens contact can be broken by the build-up of hydrostatic pressure (Fig 3); intra-ocular pressure thus rises, and acute glaucoma ensues. Viewing television from a normal distance in a dimly lit room whilst wearing distance spectacle correction does not stimulate accommodation. This results in prolonged physiological pupillary dilatation, and allows the establishment of an attack of angle closure in suseptible individuals.

The fellow eye of a patient with angle closure glaucoma is usually of similar anatomical configuration to the original eye, and it has been shown that between forty and sixty per cent of fellow eyes go on to an attack of angle closure if not treated prophylactically (Ref 1). It is therefore not surprising that occasionally bilateral angle closure glaucoma occurs.

Most patients with angle closure galucoma are initially treated by reducing the intra-ocular pressure medically. Bilateral peripheral iridectomies are then performed either surgically, or with a laser. This has the effect of bypassing the normal aqueous drainage route through the pupil.

We would like to thank Mrs. G. Bennerson for the illustrations and Ms. K. Minogue for her permission to report the cases.

### REFERENCES

1. PHILLIPS, C. I. (1986) Angle closure glaucoma diagnosis and therapy. In Glaucoma Vol II pgs 453–467, Ed Cairns J. E. Grune and Stratton, London 1986.

**Figure 1**

**Figure 2**

**Figure 3**

## Figures and Tables

**Figure 1 Figure 2 Figure 3 f1:**